# Pharmacy Student Perceptions of the Roles and Attributes of Pharmacist Preceptors in Australia

**DOI:** 10.3390/pharmacy10060169

**Published:** 2022-12-04

**Authors:** Gillian J. Knott, Martina F. Mylrea, Beverley D. Glass

**Affiliations:** Pharmacy, College of Medicine and Dentistry, James Cook University, Townsville 4811, Australia

**Keywords:** preceptor training, role model, student perceptions, assessor, mentor, educator, experiential placement

## Abstract

The pharmacist preceptor is key to the success of pharmacy student experiential placements with a recent focus being placed on the quality of preceptors. This study sought to investigate student perceptions of the ideal roles and attributes of pharmacist preceptors to inform the development of a preceptor training program. This was a mixed methods study using an online survey of pharmacy students from levels two to four of an undergraduate pharmacy honours program at a regional Australian university followed by qualitative, semi-structured focus group interviews. Surveys were analysed using descriptive statistics and content analysis while interviews were thematically analysed. Thirty-seven complete survey responses were received, and three student focus groups were conducted. Students identified the key role of preceptors in linking theory to practice and felt that the role of the preceptor in student assessment should focus on practical skills, such as communication and professionalism. There was overall strong agreement between the quantitative and qualitative findings on the ideal preceptor attributes with good communication, enthusiasm for the profession and the provision of clear and honest student feedback thought to be most important. Students regarded these attributes as essential for a good preceptor–student relationship to promote learning in the practice environment.

## 1. Introduction

Pharmacist preceptors play an integral role in developing the skills of pharmacy students during their experiential placements. With the significant increase in the amount as well as the diversity of experiential placements within pharmacy curricula worldwide, the issue of preceptor quality has been raised [1,2]. It has been reported that the quality of student experiential training is highly dependent on the preceptor, and there is a consensus that training and support for preceptors are essential to ensure that they are competent and prepared for their role [3,4]. However, the ideal structure and content of preceptor training programs are still under discussion. An examination of the roles, skills and attributes of the ideal preceptor is an important part of identifying preceptor training requirements. This should consider the opinions of all key stakeholders in the placement experience, which includes preceptors, academic staff and students [5,6].

A fundamental part of the preceptor’s role is to relate the theoretical knowledge learnt at university to the realities of pharmacy practice. To achieve this purpose, preceptors have many roles and responsibilities, including being role models, educators, mentors, and assessors to pharmacy students [7]. Several studies have investigated preceptor roles and attributes from the preceptor perspective [6,8,9,10,11]. Preceptors agree their main role is linking theory to practice and regard the key elements of their job as acting as a professional role model, educating and providing guidance and feedback on student tasks and activities. They perceive their role in student assessment to predominantly be the evaluation of professional behaviour and practical skills [6]. In 2019, DeAngelis reported that the essential skills of a preceptor should include professionalism, good communication and interpersonal skills [11]. Investigations of preceptor opinions have also identified some of the challenges of precepting that may need to be addressed with training, particularly regarding balancing time and workload when dealing with students and the provision of effective written and verbal feedback [8,10,12,13].

From the student perspective, research has also identified a range of ideal attributes and skills that they regard as important for preceptors to provide optimal benefit for the preceptee. This includes being a good role model, being interested in teaching, relating to students as an individual and giving good direction and feedback [3,14,15,16]. With pharmacy students being the main beneficiaries of precepting, insight into their experiences with preceptors and their opinions on roles, ideal skills and attributes is essential to provide a balanced and holistic perspective of preceptor training requirements. Therefore, this study focused on the perceptions of pharmacy students on the ideal roles of the James Cook University (JCU) preceptor and their associated skills and attributes with the aim of informing the design and development of a pharmacist preceptor training program.

## 2. Materials and Methods

### 2.1. Study Design and Setting

A diverse group of pharmacist preceptors provide experiential placement opportunities for James Cook University (JCU) BPharm (Hons) students. During their degree, each student is required to spend 600 h across various placement sites, mainly in community and hospital pharmacies and in both rural and urban areas under the guidance of a pharmacist preceptor.

An explanatory sequential mixed method design was utilized for this study, which included an online student survey and a series of student focus groups.

### 2.2. Quantitative—Survey

The survey was administered using the Qualtrics^®^ survey platform to all JCU students in levels 2, 3 and 4 of the BPharm (Hons) program, a total of 68 students. Students were invited to participate through a central pharmacy administration email which provided participant information and a link to the survey.

The survey questions were informed by the aim of the study and review of the literature to identify potential preceptor roles and recommended skills and attributes [3,5,17,18]. A process of evaluation was conducted by the researchers, and consensus was reached on the content of the final questions included in the survey to assure questions addressed the study’s aims. To start the survey, general demographic information was collected, including age, gender, year level and past placement experiences. Using a five-point Likert scale, students were asked to rate the importance of 15 preceptor roles as 1, unimportant; 2, slightly important; 3, moderately important; 4, important or 5, very important. Similarly, students were asked to rate the importance of 21 skills and attributes that a preceptor should possess. An open-response question was included for each of the Likert scale questions for students to provide additional comments if required. The survey was piloted by three health professional students for face validity and completion time with minor changes being made prior to survey distribution. The estimated completion time for the survey was 10 min. Upon completion of the survey, students were invited to participate in a focus group interview to further explore their opinions of preceptor roles and ideal attributes. Students were asked to provide their contact details for the purpose of arranging an interview. As an incentive, all students who participated in the focus group interviews received a $20 coffee voucher.

Survey responses were transferred into an SPSS^®^ file (SPSS 27 Statistics for Windows, Armonk, NY: IBM Corp) with the quantitative data being analysed using descriptive statistics and content analysis being utilized for the open-response questions. Chi-squared tests were conducted to determine any association between responses and demographic variables with significance set at 0.05. Content analysis performed on the open responses of the students about preceptor roles, skills and attributes categorized responses according to Condrey’s four main roles of a preceptor: role model, educator, mentor or assessor [7].

### 2.3. Qualitative—Focus Group Interviews

Three semi-structured focus group interviews were conducted, one for each of the pharmacy year levels (Years 2, 3 and 4), with each focus group consisting of three to four students. Students were chosen for the focus group interviews based on availability and purposive sampling to ensure a mix of male and female participants. Invitations were sent by email along with a participant information sheet. Interview questions were informed by the results of the student survey and included 4 main questions. The first two questions explored students’ opinions on preceptor roles as well as the skills and attributes of the ideal preceptor. Students were then asked to describe their past experiences with preceptors, including both their positive and negative experiences. Questions were modified for the Year 2 focus group to allow for their minimal (if any) past experience with preceptors. Interview questions were assessed by each member of the research team for face and content validity prior to the focus groups. Interviews were conducted face-to-face in the JCU pharmacy practice rooms, and prior to the commencement of the interview by the principal researcher, students were provided with a participant information sheet and asked to sign a participation consent form. Another research team member was present to take notes to understand aspects of the focus group not in the verbatim notes and to observe whether participants showed nonverbal agreement or dissent through nodding, head shaking or other body language. Notes were cleaned, completed and used for transcription after the focus group.

Focus group interviews were recorded and transcribed verbatim and then thematically analysed using the method outlined by Braun and Clarke [19] using NVivo. (NVivo; QSR International Pty Ltd., Version 12, 2018, Doncaster, Australia) Interview data were initially coded into three broad categories which included past experiences with preceptors, ideal preceptor roles and attributes and suggestions for enhanced placement experiences. Within each of these categories, themes were identified and presented in this manuscript.

### 2.4. Data Integration

Quantitative data from the survey and qualitative focus group data from the focus groups related to preceptor roles, skills and attributes were integrated reflecting Condrey’s approach to categorization of the four roles of preceptors as a role model, educator, mentor or assessor. Within these categories, data were reviewed and refined and then discussed as themes in this manuscript using a sample of illustrative quotes.

## 3. Results

### 3.1. Quantitative—Survey Analysis

Forty-two student survey responses were received, with 37 being complete, giving a response rate of 54.4%. Most students (73.0%) were 22 years of age or younger, and 73.0% were female, which reflects the current gender distribution of pharmacy students at JCU. All year levels were represented in the responses which include 29.7% of level two, 43.3% of level three and 27.0% of level four students. A total of 81.1% of students had attended at least one previous experiential placement in either a community pharmacy (67.6%) or a hospital pharmacy (54.0%).

**Figure 1:**
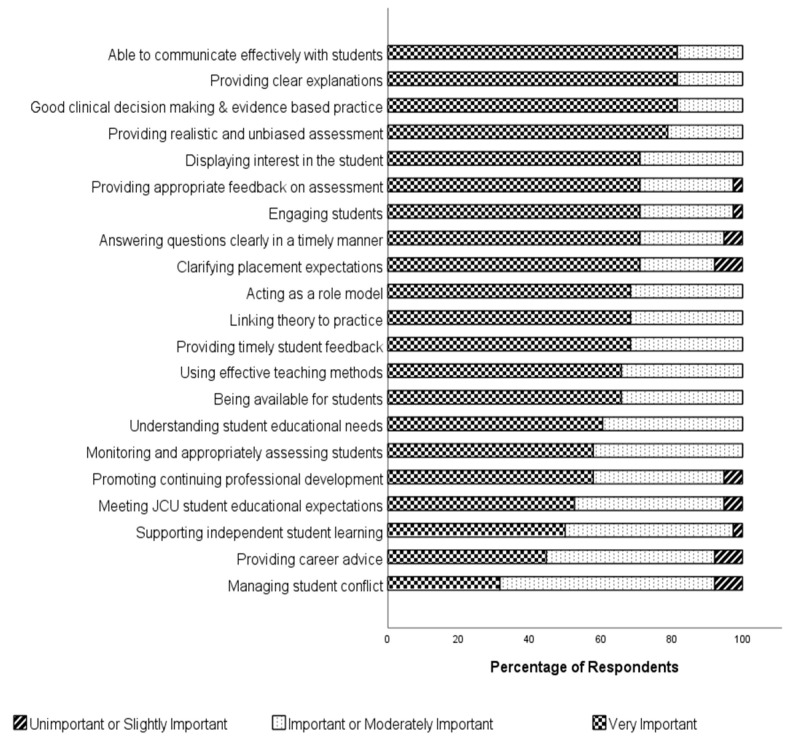
Student Opinions on Importance of Preceptor Roles (*N* = 37).

Students were asked in the survey about the importance of a range of preceptor roles, and the results are shown in Figure 1. Of the 21 potential roles for preceptors, students perceived the three most important to be communicating effectively with students, providing clear explanations and demonstrating good decision making and evidence-based practice, with all three of these roles being considered very important to 81.6% of respondents. The area of assessment was also important; they valued the provision of realistic and unbiased assessment (79.0% considered this very important) as well as appropriate feedback on assessment (71.1% considered this very important). Of least importance were the preceptor’s role in managing conflict and the provision of career advice, with 31.6% and 44.7% of participants regarding these roles as very important.

**Figure 2:**
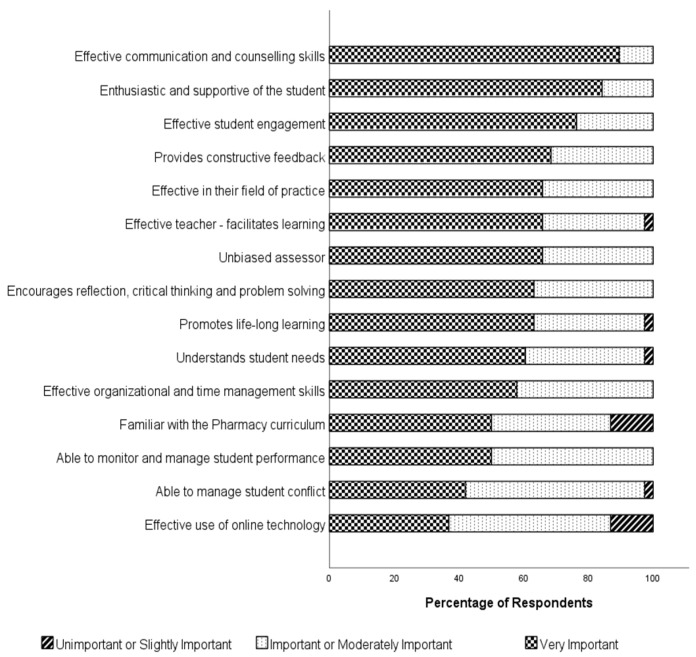
Student Opinions on Importance of Preceptor Skills and Attributes (*N* = 37).

The survey also investigated student opinions on the ideal skills and attributes of a pharmacist preceptor. The results for this question are provided in Figure 2. Of the 15 skills and attributes of the ideal preceptor, the three most important were revealed as being an effective communicator and counsellor (89.5% considered this very important), being enthusiastic and supportive of the student (84.2% considered this very important) and engaging effectively with the student (76.4% considered this very important). The least important skills were their ability to effectively use online technology (36.8% considered this very important) and their ability to manage student conflict (42.1% considered this very important). Familiarity with the pharmacy curriculum was also relatively low in importance.

### 3.2. Qualitative—Content Analysis

Content analysis of the open-ended questions in the survey revealed that the mentoring role of a preceptor was particularly highly valued with 16 comments provided. Their roles as educators and role models were also considered to be important (10 and seven comments, respectively), with only three open comments on the preceptor’s role as an assessor. Ideal attributes described by students included being encouraging, supportive, motivating, engaging and enthusiastic. Students also described a range of important preceptor skills, including effective communication, good clinical knowledge, being able to explain tasks clearly and being able to provide appropriate feedback on assessment. Table 1 shows the themes identified from the content analysis, and Table 2 provides a selection of illustrative quotes relating to these themes.

### 3.3. Quantitative Survey—Identifying Correlations between Variables

A Chi-squared test of independence showed that there was no association between student age, gender or previous placement experience and their opinions on the roles and ideal skills and attributes of a preceptor. However, there was a positive association between the student year level and the importance of preceptor familiarity with the JCU pharmacy curriculum, with this being more important in the later years of the course, χ^2^ (2, *N* = 37) = 8.2, *p* = 0.017. There was also a significant association between student year level and the preceptor’s role in meeting JCU student educational expectations, χ^2^ (2, *N* = 37) = 6.7, *p* = 0.036.

### 3.4. Qualitative—Focus Group Interview Analysis

#### 3.4.1. Experiences with Preceptors

Based on the number of student responses to focus group participation, it was decided to conduct three focus groups. All three Year Two students who agreed to participate were invited, as were the three Year Four students. While four students were invited to participate in the Year Three focus group, one student did not present on the day; therefore, this focus group also consisted of three students. Of the nine students involved, seven were female and two were male.

Focus group students discussed both positive and negative experiences with preceptors. Students were appreciative of their preceptor sharing their knowledge, taking the time to explain tasks and procedures to them and providing a range of opportunities to participate in practice. Preceptor skills and qualities that were highly valued during placement included flexibility in their teaching style, good organizational skills, enthusiasm and a recognition of the student’s prior knowledge and experiences when interacting with them.

*“… overall really positive … they were good at letting me have a go at things but then also, at certain times, being able to … step in and either take over, or just basically model how to do something”*

(Year Four Male)

One area of negativity was the consistency of information provided by the preceptor, with some preceptors taking opportunities to communicate with students and others relating poorly, which resulted in a variable student experience and led to some students being disadvantaged.

*“I guess [I would like] uniform training for all students, so some don’t get a more in-depth description [explanation] in one group, causing the other group to be at a disadvantage.”*

(Year Two Male on a group placement)

While overall preceptors were found to be enthusiastic, several students mentioned that their preceptor had a negative attitude and lacked motivation towards their job, which was thought not to contribute to student motivation or reflect well on the profession. The absence of their designated preceptor from the placement had implications, impacting the provision of informed and student-specific assessment and feedback. Several students identified the hospital environment as a particular problem with the use of multiple preceptors.

*“... You have your preceptor meeting you the first day and then you see them in and out of the hospital but you’re not actually working with them, it’s not until the last day where it’s like ‘here’s your feedback’ and it’s not a true reflection of what you have done.”*

(Year Four Female)

#### 3.4.2. Preceptor Roles, Skills and Attributes

Students identified a range of roles, skills and attributes of preceptors as important. As role models, they felt that preceptors were examples of professionals in their chosen careers and, therefore, should display behaviours expected of them in the future.

*“You look up to them. You want to develop the same techniques that they do, you want to aim to be as good as they are when you are practicing …”*

(Year Two Female)

Students felt that it was important that preceptors act as guides or mentors and establish a relationship with the student, which would make them feel comfortable with asking questions.

“… establishing a good relationship between the pharmacist and the students on placement … because if you have a good relationship … then you’ll feel more comfortable in asking questions …”

(Year Two Female)

*“I think leadership is important … Being able to manage different people and know how they interact …, knowledge is very important and also the ability to give good feedback …, constructive criticism is important, I know that they probably think that you don’t want to hear it but that’s how you learn, …”*

(Year Three Female)

In terms of their educational role, students identified that they wanted preceptors to make the link between theory and practice and to focus on teaching the ‘hands-on’ skills that students may not have covered so well at the university. They also suggested that it was important that preceptors understood the pharmacy curriculum and therefore the level of the student that they are precepting. Several students highlighted that some preceptors had unrealistic expectations of them. It was also felt that some preceptors were using the student as a replacement for staff on leave, which was not the purpose of an experiential placement.

*“Maybe if the preceptors were in touch with our curriculum … then they can help relay back to assessing and using that practical setting to apply the knowledge and skills that we learn in classes.”*

(Year Two Female)

*“Yes, I think, just them [preceptors] knowing what level we are at and where we’re expected to be … rather than them comparing us to maybe … their interns …”*

(Year Three Female)

As preceptor expectations may vary between placements, it was suggested that preceptors should ensure that they outline their expectations at the beginning of placement so that students are aware of what is required of them.

*“Probably another thing is to outline their expectations, because every pharmacist has a different way of doing things, different expectations of the level and quality of work … so … it’s important for them to show you at the beginning what they expect of you.”*

(Year Four Female)

Students were asked about the role of a preceptor in assessment and feedback. It was felt that preceptors were more suited to the assessment of practical skills, such as communication and professionalism. Several students felt that their pharmacists were constantly assessing them informally, as they were always actively listening and providing feedback, which was felt to be of benefit to student growth and development.

*“In terms of assessing, I think students learn more when they are in a practical setting. I personally learn better in a practical setting, and I think it’s important that preceptors assess that for us.”*

(Year Two Female)

*“ …. in terms of assessment… there’s assessment from the Uni’s end, which is ‘are you satisfactory or not’ … there’s some grading within that but ultimately it accounts for such a small amount of the grade. So I think more assessment in terms of …. how can you professionally improve … communication skills etc… you know, skills assessment in terms of real-life skills, not necessarily in terms of a grade.”*

(Year Four Male)

Finally, the overall importance of good communication skills was a universal comment from students.

*“I think communication is a really important part … and being able to work well in a team—essentially that is a big part of pharmacy, regardless of where you end up … you need to be able to have those skills … and be able to relay that to a student …”*

(Year Four Female)

#### 3.4.3. Suggestions for Improvement

Students identified the need for preceptor training or continuing professional development to improve the consistency of the placement experience.

*“… if you have one preceptor that’s really great at explaining and elaborating on points and one that’s not so much, then the students in that group may not benefit as much …”*

(Year Two Female on a group placement)

*“If the preceptors themselves don’t feel that confident in that teaching setting, then probably you need to do some sort of professional development like there already is that pharmacists have to do that [CPD]…….”*

(Year Two Female)

The expectations of preceptors were thought often to be unreasonable; therefore, students recommended that some expectations are set by the university for the student.

*“Our intern had 3 days off for intern training and for those three days they did not replace the intern, so I wasn’t considered an extra, I was a staff member then. ….. and that was in the first week, so when you’re new to a pharmacy and they’re expecting you to be doing all the jobs that an intern was doing when they’ve been there for months….”*

(Year Three Female)

*M1 … it’s possible that you can go on a placement that the preceptors might assume that you already know what you are doing, but then they’ve got an expectation about what they want you to do so I think it’s important that … even for a few counsellings … that they actually model what their expectations are to you ….”*

(Year Four Male)

There was much discussion about preceptor evaluations, which were often thought to be inconsistent with student performance, with preceptors not being discerning with regard to their evaluations. It was felt to be important to not only receive regular feedback but also honest and realistic feedback, whilst also being perceptive and considering individual student sensitivity.

*“… I think there’d probably need to be better standards … because with the [student] evaluation that they did at the end, results were quite varied … but it just seems like maybe there is inconsistency with how they evaluate you.”*

(Year Three Female)

In the Year Four focus group, it was suggested that, in the same way that preceptors provide feedback on their students, an opportunity for students to give feedback on their preceptors should be provided, which was reinforced by all other group members.

### 3.5. Preceptor Roles, Skills and Attributes—Data Integration

Condrey’s educational model categorized the four roles of the preceptor as a role model, educator, mentor and assessor.

As role models, students in the survey identified the importance of preceptors demonstrating good communication skills, professional behaviour and clinical knowledge. This was reinforced by the focus group participants who looked to preceptors to exemplify how they should practice and behave in the future.

As a mentor, students in the survey felt that preceptors should be engaging, enthusiastic, motivating and supportive. They also should be understanding and approachable. Further to this, focus group participants recognized the importance of establishing a relationship with the student, having leadership skills and being able to manage people.

According to the survey, the role of the preceptor as an educator focuses on understanding the purpose of the placement, setting expectations, providing clear explanations and establishing a safe learning environment. In the practice setting, students in the focus group additionally discussed the importance of providing ‘hands-on’ activities for students, linking theory to practice and having an understanding of the university curriculum to assist in identifying individual student needs.

As an assessor, the preceptor role was identified in the survey as providing realistic and unbiased assessment and appropriate feedback on that assessment. Assessment requirements should be clear, and feedback on assessment should include advice on how to improve in the future. Focus group students felt that preceptors were more suited to the assessment of practical skills and that feedback on assessment should be honest, realistic and constructive. They also felt that regular informal feedback on everyday tasks was important for student growth and development.

## 4. Discussion

This study provides valuable insight into the perceptions of JCU pharmacy students on the roles and ideal skills and attributes of pharmacist preceptors. Students attached importance to the role of the preceptor in providing real-life ‘hands-on’ pharmacy experiences and linking theory to practice. In terms of ideal skills and attributes, the importance of having a good preceptor–student relationship was highlighted, with emphasis on clear and effective communication, enthusiasm for precepting and a consistent and realistic approach to assessment and feedback.

Past research has indicated that some of the most significant student indicators of preceptor excellence include interest or enthusiasm for teaching, relating to students as an individual and good organization skills [3,16]. These outcomes align with those of this study, with students reportedly being grateful to have preceptors who were enthusiastic, well-organized and had a flexible approach to teaching.

Conversely, students were critical of preceptors who were unmotivated, with some of their negative experiences relating to the poor attitude and lack of interest of some pharmacist preceptors as well as inconsistencies in the provision of advice and feedback. In 2019, DeAngelis looked at preceptor perceptions in a range of preceptor roles or tasks based on their importance, degree of difficulty and frequency. It was identified that while the role of motivating students to be active participants was an important and frequent task of preceptors, it was also regarded as a difficult task [11]. From the student perspective, the importance of good communication between preceptor and student is well documented, with students regarding the establishment of a meaningful preceptor–student relationship as important to facilitate and nurture student development and encourage independent learning [16,17].

The educational model outlined by Condrey identifies the four main roles of preceptors as role models, educators, mentors, and assessors [7]. Through these four roles, the preceptor can fulfil their key responsibilities in linking theory to practice. According to Bochenek, the main attributes of an effective role model are being a good communicator and being knowledgeable [14]. Students in this study felt that preceptors should set an example and provide a standard for them to achieve, particularly in terms of good clinical decision making and evidence-based practice. O’Sullivan et al. similarly found that students would like their preceptors to demonstrate expertise in their practice and be able to use their communication skills to stimulate discussions with students and provide effective feedback [16].

As an educator, students in the focus groups identified the need for preceptors to be ‘in touch’ with the JCU pharmacy curriculum while both the survey and focus groups found that students placed importance on their preceptors providing clear explanations and having realistic expectations. Knowledge of the curriculum would allow the preceptor to gauge the level of experience of the student and thus provide appropriate learning opportunities as well as enable preceptors to have realistic expectations of their students. O’Sullivan et al. found that students liked their preceptors to be able to tailor their teaching to meet student needs and interests as well as provide clear feedback to students, which will encourage the development of critical thinking and aid in problem solving. They also identified the importance of preceptors being clear in their expectations [16]. However, students in our study also noted that preceptor expectations of students varied with the individual preceptor, which highlights the need for training and appropriate competency standards to provide a consistent level of precepting [20,21,22]. The inclusion of educational training for preceptors is supported by Bochenek, who found that while preceptors of pharmacy residents were thought to be effective as role models and mentors, they were less effective in their teaching and clinical skills [14]. Furthermore, a study by AlArifi on preceptee satisfaction with preceptors of Pharm D students in Saudi Arabia found that while the majority of students believed their preceptors to be knowledgeable, less than half of the students were satisfied with their preceptors’ clinical teaching skills. [23]

Both survey and focus group results indicate that the mentoring role of the preceptor was of high importance to students, with significant value placed on establishing a strong student–preceptor relationship. This focus on the mentoring role of the preceptor is highlighted by Young et al. in 2014, who found that personal attributes rather than knowledge or credentials were of greater importance to student pharmacists as a measure of preceptor excellence [3].

The mentoring role includes managing student conflict, despite this being an area considered in this study to be of relatively low importance to students. Kendrick et al. found that conflict in experiential education was thought to be a common occurrence and was reported by both preceptors and learners [24]. They noted that preceptors were more likely to take the initiative to resolve conflict, which may reflect the unequal power relationship between preceptor and learner and may be the reason why this topic may not be seen as important to students. It was determined in this study that preceptors lacked overall confidence in managing conflict and that training in this area would be important for both preceptors and learners, with studies by Assemi et al. and Phillips et al. also reflecting this view [13,25].

The role of the preceptor in assessment during experiential placement is unclear. Students felt that the preceptor’s role in formal assessment was of the least importance and should be limited to the assessment of practice-based tasks, such as communication and professionalism. In Australia, Kirshbaum reported that many universities do not require preceptor feedback or assessment in the evaluation of student placement, and this may be the reason for the low ranking of importance from students [26]. A key issue raised was in the consistency of assessment and feedback, where it was felt that some preceptors were not discerning, which led to students receiving a result that did not appropriately reflect their performance. Similarly, in the provision of feedback, student focus groups identified a lack of consistency in the amount and quality of feedback provided by individual preceptors. In the provision of student feedback in the clinical environment, it is recommended that it is constructive, realistic, meaningful, honest and unbiased [27,28,29]. In Young’s student survey, it was found that being readily available for questions and giving good direction and feedback was an important indicator of preceptor excellence [3]. It was further noted by Sonthisombat that many preceptors focused on the provision of clinical information rather than understanding the learning styles of the student, which may be detrimental to the quality of their feedback [15]. Danielson et al. suggested that maintaining the quality of student assessment was a concern for many preceptors [30]. Lucas et al. identified the difficulties involved in providing consistency in student evaluation and feedback processes, particularly given the variation in placement environments. However, they also considered it desirable to have some consistency in these processes [10].

Limitations of this study include the fact that it was conducted at a single university, which may not be representative of the wider population of pharmacy students. While the sample size was relatively small, the response rate of more than 50% in the survey and the nature of the respondents in terms of gender and year level ensured that a representative sample of the student population at this regional university was included, with the mixed methods approach assisting by providing both breadth and depth of information. There was some potential for student response bias with the principal researcher being also involved in student teaching. However, this bias was thought to be minimal as the students stood to benefit from the study. Survey and interview questions were also carefully worded to ensure that researcher bias was minimized.

## 5. Conclusions

This study has explored the roles and ideal attributes of pharmacist preceptors from the viewpoint of pharmacy students. With students being the predominant beneficiaries of the efforts of preceptors, their perceptions of roles and attributes to inform preceptor training are important for the optimal development of training programs. While clinical knowledge is an essential attribute for all preceptors, students perceived that good communication and an interest and desire to teach were more important overarching skills to possess to achieve preceptor excellence. Preceptor training that has a focus on building good preceptor–student relationships will assist in meeting the challenges of developing competent and independent pharmacy graduates in the future.

## Figures and Tables

**Table 1:** Content Analysis—Student opinions of important preceptor skills and attributes.

Role	Themes
Role Model	Professional behaviour Good communication (2) * Patient centred care Managing difficult patients Good clinical knowledge Realistic student experiences
Educator	Safe learning environment Providing hands-on activities (3) * Explaining tasks clearly Explaining the reasons why we perform tasks Understanding the student Patience Understand their educational role Providing opportunities for student questions
Mentor	Engaging with the student (2) * Motivating Enthusiastic about placement (2) * Supportive (4) * Approachable Friendly Understanding (2) * Being available for questions
Assessor	Clear assessment requirements Good feedback on assessment (2) *

* Indicates the number of quotes greater than one which identified this theme.

**Table 2:** Content Analysis—Selected student quotes on important preceptor skills and attributes.

Role	Quotes
Role Model	“Providing realistic experiences to students in the pharmacy” “Model professional behaviour and mutual respect….” “Model patient-centred care approaches as much as practicable” “That they know what they are talking about” “Show a good example of how to talk with patients and handle difficult patients” “Show a good example of how to talk to doctors and other health professionals”
Educator	“Providing a safe and encouraging environment to facilitate learning and opportunities for students to ask questions which may further learning” “Generate physical activities to allow the student to engage with the profession during their placement” “Student involvement, rather than just observing- especially in Third and Fourth year” “Taking time to explain to students how to do tasks clearly and methodically, why we do certain tasks and being patient” “Good preceptors realise that students aren’t the finished product yet and don’t get frustrated if they have some gaps in knowledge” “Preceptors should be understanding towards what the students are there to do. They should not be treated as if they are a staff member of that pharmacy.”
Mentor	“Encourage students to see the value in all tasks in the pharmacy, and to engage with them in a positive manner (even unglamourous tasks)” “Motivators of students to improve and be a better version of themselves” “Engaging and genuinely interested in helping students to learn about being a pharmacist” “Enthusiasm for student placement and taking the time for education opportunities’ “A supportive and approachable preceptor is important in order for students to feel comfortable asking them for guidance or questions” “It is also important for the preceptor to be present at the placement every day of the placement unless sick or it is an emergency. The responsibility of a preceptor should not fall onto another pharmacist that did not initially agree to being a preceptor” “Should be relatable so that students feel comfortable approaching for guidance’ ‘Be more friendly and understanding when students ask questions about things they may not have heard or understood” “Being available to answer questions and provide help when required is probably the most important trait”
Assessor	“Explain requirements for assessments clearly so there is no confusion” “Should have the ability to justify and give tips for improvement based on marks and assessment they provide”

## Data Availability

This study did not report any data.
